# Identification of Plasma Exosomes hsa_circ_0001360 and hsa_circ_0000038 as Key Biomarkers of Coronary Heart Disease

**DOI:** 10.1155/2024/5557143

**Published:** 2024-03-26

**Authors:** Wan Zhang, Jiasen Cui, Li Li, Ting Zhu, Zhenyu Guo

**Affiliations:** ^1^Department of Vascular Surgery of Huadong Hospital, Affiliated to Fudan University, Shanghai 200040, China; ^2^Department of Vascular Surgery of Zhongshan Hospital, Affiliated to Fudan University, Shanghai 200032, China

## Abstract

**Background:**

Coronary heart disease (CHD) is the leading cause of death and disability worldwide. Accumulating evidence reveals that atherosclerosis (AS), characterized by systemic, chronic, and multifocal disease, and is the primary pathological basis of cardiovascular diseases, including CHD. However, the molecular underpinnings of CHD are still far from well understood. Our study attempted to identify aberrant plasma exosome-derived circRNAs and key exosomal circRNA biomarkers for CHD.

**Methods:**

The expression profiles of mRNAs, circRNAs, and lncRNAs in the blood exosomes of CHD patients and healthy controls were obtained from the exoRBase database. The corresponding miRNAs of the differentially expressed mRNAs, circRNAs, and lncRNAs were predicted via ENCORI and the miRcode database. LncRNAs/circRNAs and mRNAs with the cotargeted miRNAs were selected to construct an interaction network. Multiple machine learning algorithms have been used to explore potential biomarkers, followed by verification in patients with CHD using real-time quantitative reverse transcription-polymerase chain reaction (RT-qPCR).

**Results:**

Based on the cutoff criterion of *P* < 0.05, we identified 85 differentially expressed circRNAs (4 upregulated and 81 downregulated), 43 differentially expressed lncRNAs (24 upregulated and 19 downregulated), and 312 differentially expressed mRNAs (55 upregulated and 257 downregulated). Functional enrichment analysis revealed that the differentially expressed mRNAs were involved mainly in neutrophil extracellular trap (NET) formation and the nucleotide-binding oligomerization domain- (NOD-) like receptor signaling pathway. Further analysis revealed that the DEGs in the circRNA/lncRNA-miRNA-mRNA interaction network were closely related to lipid and atherosclerotic signaling pathways. Hsa_circ_0001360 and hsa_circ_0000038 were identified as potential biomarkers for CHD based on three machine learning algorithms. The relative expression levels of hsa_circ_0001360 and hsa_circ_0000038 were significantly altered in plasma exosomes from patients with CHD. ROC curve analysis revealed that the areas under the curve (AUCs) were 0.860, 0.870, and 0.940 for hsa_circ_0001360, hsa_circ_0000038, and the two-gene combination, respectively.

**Conclusion:**

The circRNA/lncRNA-miRNA-mRNA interaction network might help to elucidate the pathogenesis of CHD. Hsa_circ_0001360 combined with hsa_circ_0000038 might be an important diagnostic biomarker.

## 1. Introduction

Coronary heart disease (CHD) is a classic type of cardiovascular disease characterized by a decreased oxygenated blood supply to the heart. CHD, with its associated complications, contributes to multiple morbidities and mortality worldwide [1, 2]. Epidemiological studies revealed that more than 16 million adults suffered from CHD in the United States in 2016 [3], and this number is predicted to rise dramatically due to the increasing aging population. These conditions place a great burden on patients' physical and psychological health, as well as on society's healthcare resources [4]. Therefore, current guidelines place great emphasis on early detection and preventive strategies for CHD.

Atherogenesis serves as the primary pathological basis of CHD [1]. Accumulating evidence has revealed that atherosclerosis (AS) is a systemic, chronic, and multifocal disease initiated by the retention and accumulation of lipid particles. In addition, intimal injury, platelet activation, inflammatory cell infiltration, and vascular smooth muscle cell migration are involved in the initiation and progression of this pathological process [5–7]. However, based on the high prevalence of AS, we believe that there is still novel insight to be gained regarding AS.

Exosomes are membrane-bound extracellular vesicles that are characterized by nanosized endocytic vesicles secreted by cells with a diameter of 30∼100 nm; they contain biological molecules, such as noncoding RNA, mRNA, and proteins, that are derived from the corresponding cells [8, 9]. Noncoding RNA refers to RNA that does not encode proteins, including circular RNAs (circRNAs), long noncoding RNAs (lncRNAs), microRNAs (miRNAs), and RNA with unknown functions, and it plays an important role in bioprocesses, including cell proliferation, differentiation, and migration [10, 11]. These bioactive molecules enable exosomes to function as potent communicating intermediaries that can transmit genetic information and subsequently participate in physiological and pathological processes [12, 13]. Recent studies have also shown that exosomes play a crucial role in intercellular communication during the inflammatory response, which implies a close relationship between exosomes and AS [14–16]. However, to our knowledge, the exosome-related noncoding RNAs involved in the pathogenesis of AS have not been fully elucidated to date.

In the present study, we constructed a circRNA/lncRNA-miRNA-mRNA network in blood exosomes derived from patients with CHD based on a biological database. Next, we validated the aberrant circRNAs in the network in patients with CHD by real-time quantitative reverse transcription-polymerase chain reaction (RT-qPCR). A receiver operating characteristic (ROC) curve was plotted to analyze the clinical implications of these results. We attempted to identify novel exosomal circRNA diagnostic biomarkers for CHD and contribute to our understanding of the pathogenesis of AS.

## 2. Materials and Methods

### 2.1. Ethics Statement

The study was approved by the ethics committee of Huadong Hospital, affiliated with Fudan University (2022K171). All procedures conformed to the principles outlined in the Declaration of Helsinki. All patients provided written informed consent before enrollment in the study.

### 2.2. Data Source and Preprocessing

The exoRBase is a repository of human blood exosome-derived long RNA species, including circRNAs, long noncoding RNAs (lncRNAs), and long noncoding RNAs (mRNAs). Following detection by normalized RNA-sequencing data analysis, exoRBase was used to collect and integrate RNA expression profiles from healthy individuals and patients with corresponding diseases [17]. The RNA expression profiles of normal individuals (*n* = 32) and patients with CHD (*n* = 6) were downloaded from the exoRBase database, followed by background correction and data normalization in R software (version 4.0.2).

### 2.3. Screening of Differentially Expressed Genes, circRNAs, and lncRNAs

The limma package was applied to the RNA-sequencing data analysis to remove batch effects and filter differentially expressed genes, circRNAs, and lncRNAs by comparing the CHD group to the control group. Long RNA species with *P* values less than 0.05 were considered differentially expressed genes (DEGs), circRNAs (DE-circRNAs), and lncRNAs (DE-lncRNAs).

### 2.4. Prediction of Potential miRNAs for Differentially Expressed Genes, circRNAs, and lncRNAs

ENCORI, the encyclopedia of RNA interactomes (https://starbase.sysu.edu.cn/), was applied to the prediction of potential miRNAs for differentially expressed genes and circRNAs. The predicted miRNAs of genes were further validated by the TargetScan and miRanda databases. The overlapping miRNAs were selected for subsequent analysis. The miRcode database (https://www.mircode.org/), which provides transcriptome-wide microRNA target prediction information, was used to explore lncRNA-miRNA interactions. LncRNAs/circRNAs and mRNAs with the cotargeted miRNAs were selected to construct an interaction network, and the results were visualized with Cytoscape 3.8.1.

### 2.5. Functional and Pathway Enrichment Analysis

Gene Ontology (GO) terms and Kyoto Encyclopedia of Genes and Genomes (KEGG) analyses were further conducted to gain deeper insight into the functions and pathway enrichment of the target genes. The process was implemented by the “clusterProfiler” (version 3.18.1) package [18].

### 2.6. Screening and Verification of Diagnostic Markers

Least absolute shrinkage and selection operator (LASSO), random forest (RF), and support vector machine recursive feature elimination (SVM-RFE) methods were used to identify key biomarkers for CHD. The LASSO algorithm is a regression analysis method that performs variable selection by exerting a contractile penalty function on variables to enhance the prediction accuracy and interpretability of the statistical model. This process was implemented via the “glmnet” package [19]. In addition, the RF algorithm and SVM-RFE were applied with the “RandomForestSRC” and “e1071” packages, respectively. Further receiver operating characteristic (ROC) curve analysis was conducted to evaluate the diagnostic efficacy of the biomarkers.

### 2.7. Patient Sample Collection

Patients with CHD (CHD group, *n* = 10) were recruited between August 2021 and November 2021. Patients with CHD were all diagnosed based on computed tomography angiography. Age-matched healthy individuals (those without cardiovascular disease, diabetes, or hyperlipidemia; *n* = 10) were recruited as the control group. The exclusion criteria included [1] a history of tumor or connective tissue disease, [2] steroid use within 3 months, [3] recent infection, and [4] failure to sign the informed consent form.

### 2.8. Exosome Isolation

Blood samples were collected from both groups at 8:00 a.m. after they had fasted for at least 8 hours. Fresh blood samples were subjected to centrifugation at a speed of 3000 × *g* for 10 minutes at 4°C. The supernatant was subsequently transferred to another tube, and plasma exosomes were isolated using a Hieff™ Quick exosome isolation kit (for Serum/Plasma, Yeasen Biotech Co., Ltd., Shanghai, China) according to the manufacturer's instructions. The morphological characteristics and size distribution of the plasma-derived exosomes were further assessed by transmission electron microscopy and nanoparticle tracking analysis as previously described [20].

### 2.9. RT-qPCR Assays

Total RNA was extracted from plasma exosomes with TRIzol reagent (Invitrogen, Carlsbad, CA). Subsequent reverse transcription of circRNA was conducted with the random primer N6 from the Hifair® III 1st Strand cDNA Synthesis Kit (gDNA digester plus, Yeasen Biotech Co., Ltd.) following the manufacturer's instructions. The comparison of the expression levels of target circRNAs was performed by RT‒qPCR based on the ABI QuantStudio 5 Real-Time PCR System (Thermo Fisher Scientific, Waltham, MA, USA), with GAPDH as an internal reference. The corresponding primers were designed with circPrimer 1.2 (https://www.bioinf.com.cn/, Supplementary Table 1).

### 2.10. Statistical Analysis

Statistical analysis was performed with the Student's *t*-test or the *χ*^2^ test as appropriate using SPSS (version 24.0). A *P* value less than 0.05 was considered to indicate statistical significance.

## 3. Results

### 3.1. Screening of Differentially Expressed Genes, circRNAs, and lncRNAs

**Figure 1:**
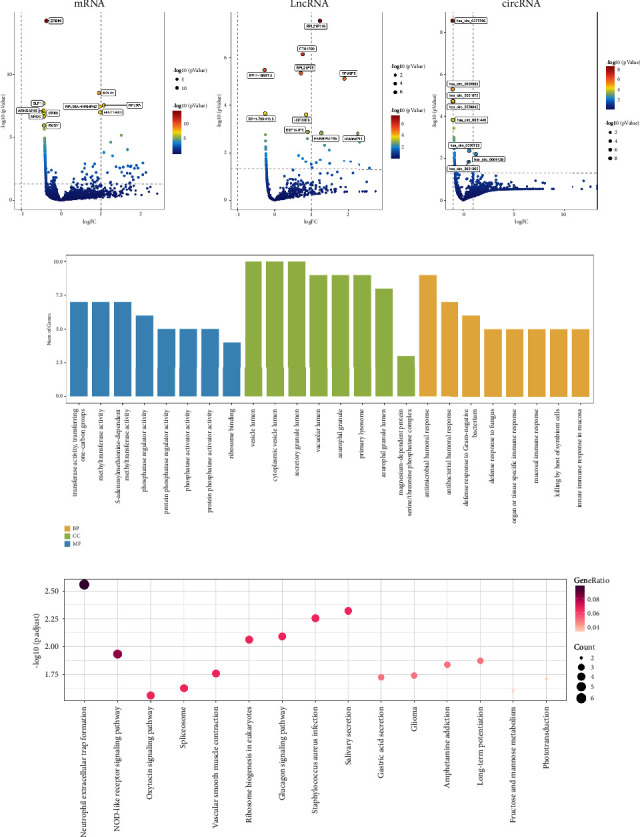
Differentially expressed circRNAs, lncRNAs, and mRNAs in blood exosomes derived from the normal and CHD groups. (a) Differentially expressed circRNAs, lncRNAs, and mRNAs. (b) Gene Ontology (GO) analyses of differentially expressed genes. (c) Kyoto Encyclopedia of Genes and Genomes (KEGG) analyses of DEGs.

After expression profile normalization, the circRNA and gene expression matrix derived from 32 normal and 6 CHD samples was used to screen differentially expressed RNAs (DEGs) (Figure 1(a)). Based on the cutoff criterion of a *P* value less than 0.05, we identified 85 differentially expressed circRNAs (4 up- and 81 downregulated), 43 differentially expressed lncRNAs (24 up- and 19 downregulated), and 312 differentially expressed genes (55 upregulated and 257 downregulated). GO enrichment analysis revealed that the differentially expressed mRNAs were enriched mainly in biological processes such as the inflammatory immune response (Figure 1(b)). Based on KEGG enrichment analysis, DEGs were mainly involved in neutrophil extracellular trap (NET) formation and the nucleotide-binding oligomerization domain (NOD)-like receptor signaling pathway (Figure 1(c)).

### 3.2. Construction of the circRNA/lncRNA-miRNA-mRNA Network

**Figure 2:**
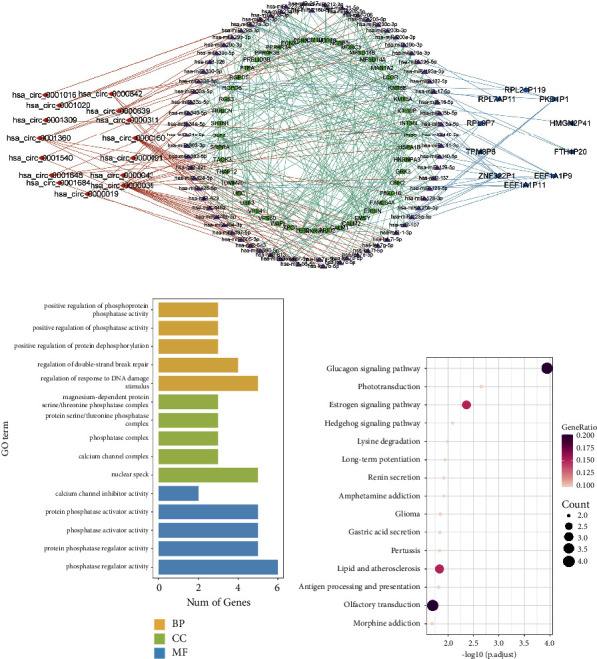
Functional enrichment analysis of DEGs in the circRNA/lncRNA-miRNA-mRNA network. (a) CircRNA/lncRNA-miRNA-mRNA network construction. (b) Gene Ontology (GO) analyses. (c) KEGG enrichment analyses.

First, 176 miRNAs targeting DEGs and 171 miRNAs with binding sites on DE-circRNAs were predicted by the ENCORI and TargetScan databases. In addition, 166 miRNAs that included binding sites of DE-lncRNAs were obtained from the miRcode database. The overlaps of the predicted miRNAs were selected based on a competitive endogenous regulatory network. Finally, the circRNA/lncRNA-miRNA-mRNA regulatory network of blood exosomes involved in CHD was constructed with Cytoscape 3.8.1. As a result, 72 overlapping miRNAs were selected, and the network was visualized with Cytoscape 3.8.1 (Figure 2(a)). Further analysis revealed that the DEGs in the regulatory network were closely related to lipid and atherosclerotic signaling pathways (Figures 2(b) and 2(c)).

### 3.3. Identification of Key Blood Exosome circRNAs for CHD

**Figure 3:**
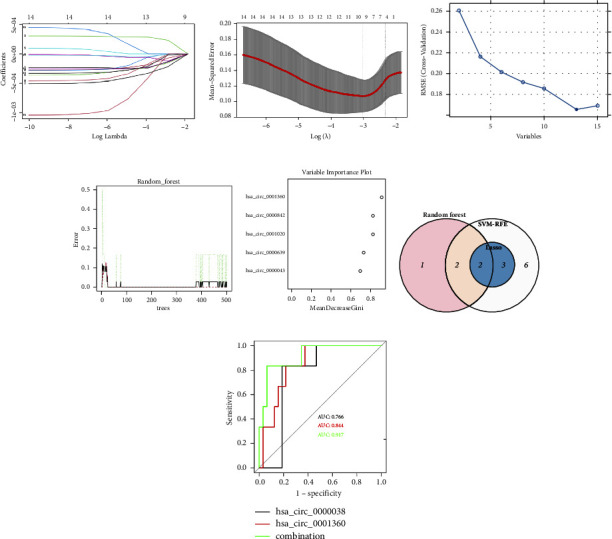
Screening and verification of key markers based on machine learning algorithms. (a and b) LASSO logistic regression analysis to identify diagnostic markers. (c) SVM-RFE algorithm for screening diagnostic markers. (d and e) RF algorithm for screening diagnostic markers. (f) Venn diagram showing the intersection of diagnostic markers. (g) ROC curve analysis.

The competitive endogenous regulatory network identified 15 differentially expressed circRNAs, including hsa_circ_0000038, hsa_circ_0001360, hsa_circ_0001020, hsa_circ_0000160, hsa_circ_0000639, hsa_circ_0001309, hsa_circ_0000019, hsa_circ_0001540, hsa_circ_0001648, hsa_circ_0000842, hsa_circ_0001016, hsa_circ_0000091, hsa_circ_0000043, hsa_circ_0001684, and hsa_circ_0000311 (Figure 2(a)). Furthermore, key biomarkers were screened from the 15 differentially expressed circRNAs via LASSO regression analysis (Figures 3(a) and 3(b)), SVM-RFE (Figure 3(c)) and the RF algorithm (Figures 3(d) and 3(e)). The 3 sets of diagnostic circRNAs overlapped, and two biomarkers were identified (Figure 3(f), hsa_circ_0001360 and hsa_circ_0000038). To better understand the diagnostic efficacy of hsa_circ_0001360 and hsa_circ_0000038, ROC curve analysis was conducted, and the area under the curve values were 0.766, 0.844, and 0.917 for hsa_circ_0000038, hsa_circ_0001360, and the two genes combined, respectively (Figure 3(g)).

### 3.4. Validation of Differentially Expressed circRNAs in Plasma Exosomes from Patients with CHD

**Figure 4:**
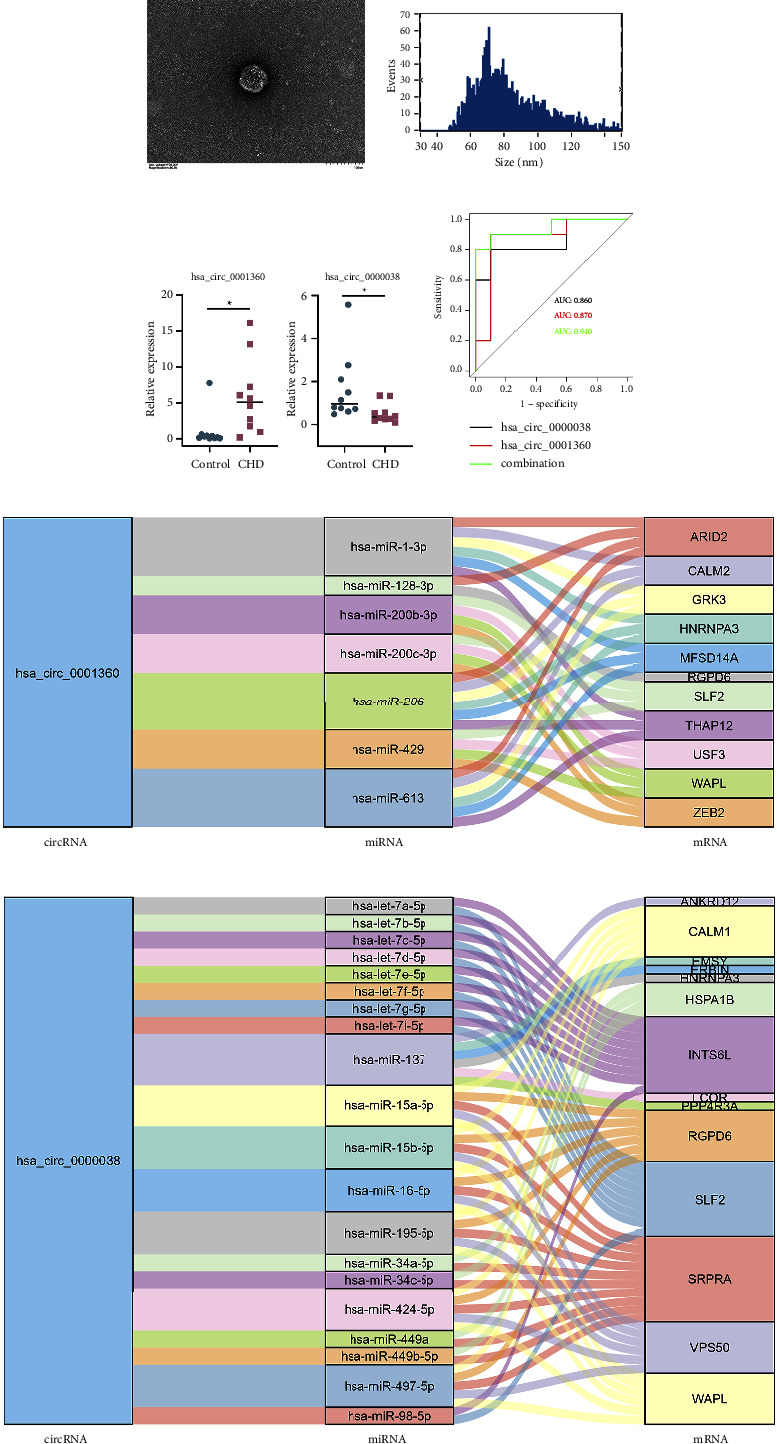
Exosome identification and key circRNA validation. (a) The morphological characteristics of the exosomes were assessed by transmission electron microscopy. (b) The size distribution of the exosomes was measured by nanoparticle tracking analysis. (c) Validation of the key circRNAs in normal and CHD samples. ^∗^indicates statistical significance, with a *P* value less than 0.05. (d) ROC curve analysis. (e and f) The downstream miRNA and mRNA networks of hsa_circ_0001360 and hsa_circ_0000038.

Blood exosome samples were collected from the control group (6 men; mean age, 68.5 y; range, 60–77 y) and the CHD group (8 men; mean age, 67.9 y; range, 60–75 y). The basic characteristics of the two groups are described in Supplementary Table 2. The morphological characteristics and size distribution of the exosomes were further assessed by transmission electron microscopy and nanoparticle tracking analysis (Figures 4(a) and 4(b)). No significant differences in morphological characteristics or exosome size were observed between the 2 groups. As shown in Figure 4(c), the relative expression level of hsa_circ_0001360 was significantly upregulated (*P* < 0.05) in patients with CHD, while the expression level of hsa_circ_0000038 was markedly downregulated. The other circRNAs obtained from 2 machine learning methods (RF and SVM-RFE, or LASSO and SVM-RFE) were also detected in clinical specimens (Supplementary Figure 1). Further ROC curve analysis was performed to evaluate the potential diagnostic value of hsa_circ_0000038 and hsa_circ_0001360 (Figure 4(d)). The areas under the curve of hsa_circ_0000038, hsa_circ_0001360, and the two-gene combination were 0.860, 0.870, and 0.940, respectively. The downstream miRNA and mRNA networks targeted by hsa_circ_0001360 and hsa_circ_0000038 are shown in Figures 4(e) and 4(f).

## 4. Discussion

Extensive studies have previously demonstrated associations among blood exosomes, AS, and cardiovascular diseases [12]. CircRNAs, characterized by a conserved ring structure and strong resistance to RNase, can act as miRNA sponges through a competitive endogenous regulatory mechanism and subsequently participate in the regulation of gene expression [10]. Recently, circRNAs derived from blood exosomes have attracted increasing attention. Due to the high prevalence of AS and CVD, circRNAs derived from blood exosomes might also play an important role in the diagnosis and treatment of CVD.

Our study comprehensively constructed a circRNA/lncRNA-miRNA-mRNA regulatory network based on the exoRBase database. We identified 85 significantly differentially expressed circRNAs, 43 significantly differentially expressed lncRNAs, and 312 significantly differentially expressed mRNAs in blood exosomes from CHD patients. The differentially expressed genes were mainly enriched in biological processes such as inflammatory immune responses, which are closely related to the pathogenesis of AS [21]. In addition, KEGG analysis revealed that the DEGs were enriched mainly in the NET formation and NOD-like receptor-mediated signaling pathways. NETs are large net-like structures composed of decondensed chromosomes and nuclear proteins, cytoplasmic proteins, and granular proteins derived from neutrophils that are capable of trapping and killing pathogens [22–24]. NOD-like receptors have also received widespread attention for their key roles in innate immune responses. Among them, NOD-like receptor protein 3 (NLRP3) is one of the key regulatory proteins of the inflammasome. In-depth analyses of AS have shown that NET formation and NOD-like rec eptor-mediated signaling pathways play important roles in promoting vascular inflammation and driving the progression of AS [25–27].

CircRNAs are covalently closed noncoding RNAs, and their expression and biological functions are gradually being revealed. Studies have shown that circRNAs can act as “miRNA sponges,” participating in the regulation of downstream gene expression by competitively binding miRNAs [28, 29]. The competitive regulatory network has been reported to have important significance in cardiovascular diseases [29]; however, the regulatory mechanisms of circRNAs in CHD have not been fully established. Based on the obtained differentially expressed RNAs and competitive endogenous regulatory network, this study selected overlapping miRNAs and further comprehensively constructed a circRNA-miRNA-mRNA regulatory network for CHD patients. Biofunctional enrichment analysis suggested that the genes in the circRNA-miRNA-mRNA regulatory network are closely related to lipid and atherosclerosis signaling pathways. The study results suggested the potential role of this circRNA-miRNA-mRNA regulatory network in the pathogenesis of CHD.

Due to the insidious progression of CHD, patients are likely to underestimate the potential harm and often have severe vascular lesions at diagnosis. Therefore, early identification of high-risk CHD patients is helpful for the prevention and treatment of CHD and its related adverse cardiovascular events, thereby reducing morbidity and mortality. Owing to the significant progress in next-generation sequencing technology, great attention has been given to circRNAs, which are involved in transcription and translation processes [30]. The covalent loop enhances the stability of circRNA and enables it to be an ideal target for potential diagnostic biomarkers. In the present study, 15 circRNAs were ultimately identified based on the circRNA/lncRNA-miRNA-mRNA regulatory network. Moreover, three machine learning methods (LASSO, RF, and SVM-RFE) showed that hsa_circ_0000038 and hsa_circ_0001360 were characteristic variables of CHD. The potential biomarkers hsa_circ_0000038 and hsa_circ_0001360 were further verified in the blood exosomes of CHD patients. Compared with that in the control group, the expression of hsa_circ_0001360 in the CHD group was markedly upregulated (*P* < 0.05), while the expression of hsa_circ_0000038 was significantly decreased (*P* < 0.05). The results of this study showed that hsa_circ_0001360 combined with hsa_circ_0000038 may be a new diagnostic biomarker for CHD.

To further explore the potential role of circRNAs in disease progression, downstream miRNAs and mRNAs targeted by hsa_circ_0000038 and hsa_circ_0001360 were screened based on the circRNA-miRNA-mRNA regulatory network (Figures 4(e) and 4(f)). Previous data have shown that zinc finger E-box binding homeobox 2 (ZEB2) is closely related to the development of cardiovascular diseases [31–33]. Recent studies have shown that ZEB2 reduces the S-sulfhydration of protein disulfide isomerase by inhibiting the cystathionine *γ*-lyase/hydrogen sulfide system, thereby inducing the initiation of aortic aneurysms and dissections [34]. Through a genome-wide association study, Cheng et al. revealed that ZEB2 is a new CHD-related gene that affects plaque vulnerability by directly regulating the epigenome [33]. In addition, some research has revealed that miR-147a regulates the formation of oxidized low-density lipoprotein-induced atherosclerotic plaques by targeting the ZEB2 gene [32]. Therefore, an in-depth exploration of the regulatory mechanism of hsa_circ_0001360 and ZEB2 will help to clarify the pathophysiological process of AS and provide new strategies for the clinical treatment of CHD.

This study has several limitations [1]. There were only 6 CHD patients in the database included in this study [2]. External validation is necessary to evaluate an existing model's performance. The study was limited by the small cohort size of a single center. A large sample analysis is needed to enhance the statistical power [3]. The morphological characteristics and size distribution of blood exosomes derived from plasma were evaluated only by transmission electron microscopy and nanoparticle tracking analysis, without the detection of characteristic exosome proteins [4]. Our study revealed that hsa_circ_0001360 and hsa_circ_0000038 were associated with only CHD; however, this finding does not prove causal. Further in vivo/in vitro experimental verification is needed to explore the role of circRNAs in the pathological process of AS and the pathogenesis of CHD. First, we identified the key cell types in which circRNAs were most abundant. Second, we validated downstream target genes and explored the role of circRNAs in the regulation of cell function and inflammatory reactions. Third, we investigated the effect of circRNAs on the development of atherosclerosis via in vivo experiments.

## 5. Conclusion

In summary, our study constructed a circRNA/lncRNA-miRNA-mRNA regulatory network of CHD patients based on the exoRBase database, followed by screening and validation of potential biomarkers through multiple machine learning algorithms and RT-PCR. Hsa_circ_0001360 and hsa_circ_0000038 could serve as key diagnostic markers in CHD patients.

## Data Availability

The raw data used in this study were derived from the exoRBase database (https://www.exorbase.org/). All the data analyzed in this study are included in this published article and its supplementary information file.

## Supplementary Materials

Supplementary Materials Supplementary Table I: Primers used for RT-qPCR the primers of circRNAs were designed with circPrimer 1.2 (https://www.bioinf.com.cn/). CircPrimer is a user-friendly tool to search, annotate, and visualize circRNAs. Additionally, circPrimer enables users to extract the spliced sequences and genomic sequences of any circRNA, including novel circRNAs. In this study, circPrimer was used to design primers for circRNAs and to determine the specificity of the circRNA primers. Supplementary Table II: Basic characteristics Supplementary Fig. 1: Expression levels of circRNAs in normal and AS groups. AS: atherosclerosis.
