# Supplementary figures and images for: Identification of Plasma Exosomes hsa_circ_0001360 and hsa_circ_0000038 as Key Biomarkers of Coronary Heart Disease
Source: Cardiol Res Pract. 2024 Mar 26;2024:5557143. doi: 10.1155/2024/5557143 (PMC10987246; doi:10.1155/2024/5557143)

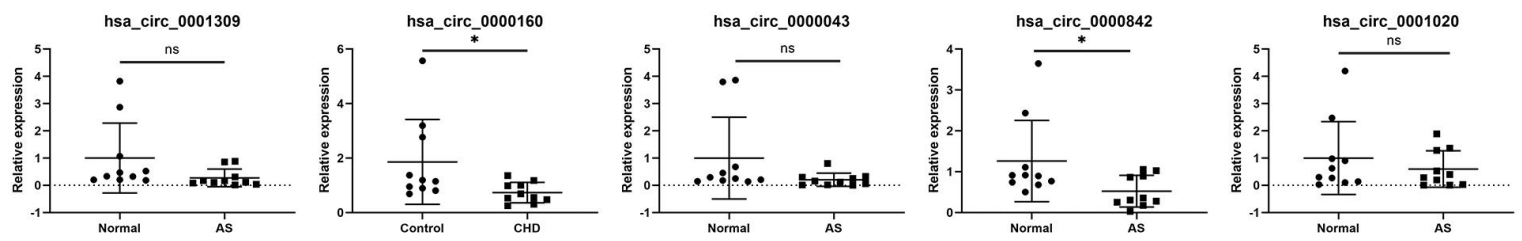

Supplementary Fig1. Expression levels of circRNAs in normal and AS groups. AS: atherosclerosis.

Supplement: Supplementary Materials — Supplementary Table I: Primers used for RT-qPCR the primers of circRNAs were designed with circPrimer 1.2 (https://www.bioinf.com.cn/). CircPrimer is a user-friendly tool to search, annotate, and visualize circRNAs. Additionally, circPrimer enables users to extract the spliced sequences and genomic sequences of any circRNA, including novel circRNAs. In this study, circPrimer was used to design primers for circRNAs and to determine the specificity of the circRNA primers. Supplementary Table II: Basic characteristics Supplementary Fig. 1: Expression levels of circRNAs in normal and AS groups. AS: atherosclerosis. [file 5557143.f1.zip › Supplementary Fig1 (1).pdf]
